# Ecology and management of the invasive land snail *Bulimulus bonariensis* (Rafinesque, 1833) (Stylommatophora: Bulimulidae) in row crops

**DOI:** 10.3389/finsc.2022.1056545

**Published:** 2022-12-20

**Authors:** Marcelo Mendes Rabelo, Marcelo Dimase, Silvana V. Paula-Moraes

**Affiliations:** Department of Entomology and Nematology, West Florida Research and Education Center, University of Florida, Jay, FL, United States

**Keywords:** peanut, soybean, tillage, sampling, trapping, management

## Abstract

Solutions for managing the growing populations of the snail *Bulimulus bonariensis* (Rafinesque, 1833) in row crops, notably peanut (*Arachis hypogaea* L.), are urgently needed in the United States. This species has become a concern to the economy and food security for infesting commercial crops in U.S. southern states. In the present study, sampling, trapping, and management strategies were investigated to support a management program for *B. bonariensis* in row crops. In addition, the preference of *B. bonariensis* for species of row crops and weeds, used as a shelter, and snail dispersal capacity were documented. The results indicated that the ideal tools for monitoring and capturing snails are beat cloth and cardboard trap, respectively. Metaldehyde 4% bait produced effective control. Tillage was tested as an alternative cultural management tactic and produced the most promising outcomes in lowering snail populations. According to snail ecological studies, peanut and soybean are the preferred crops used as shelter over cotton and corn. Among eight common winter-growing weeds, the favored non-crop host plants are cutleaf primrose (*Oenothera laciniata*) and dandelion (*Taraxacum officinale*). The snail field population tends to increase as early spring temperatures rise, with more snails becoming trapped in warm, humid conditions but not through heavy precipitation. This study provides ecology information on *B. bonariensis* and validates tactics to manage this invasive species in row crops, in an IPM approach.

## 1 Introduction

Terrestrial pulmonated gastropods, primarily known as slugs and snails, have become one of the most difficult pests to manage in agroecosystems, resulting in significant economic damage to a wide range of crops, including oilseed rape, vegetables, legumes, cereals, and fruits (1–3). Although slugs may cause economic impact in row crops, this study will focus on the invasive land snail *Bulimulus bonariensis* (Rafinesque, 1833) (Stylommatophora: Bulimulidae). *Bulimulus bonariensis*, a synonym of *Bulimulus sporadicus* (d’Orbigny), has recently caused severe problems across the southern U.S (4–6), and is native to the West Indies. These snails were first reported in Florida in the Jacksonville area in 2009 and has since spread to at least 26 counties within the state (6–10).

*Bulimulus bonariensis* is a detritivore and does not typically cause injury from plant feeding, although it will occasionally feed on plants when no other food is available, and high populations can compromise seedling development (11–16). The major issue concerning *B. bonariensis* infestation is the risk of food contamination in crops, such as peanut (*Arachis hypogaea* L.). Current updraft air column harvest systems cannot separate the snails from peanut kernels, as both can have similar size, color, and weight. Aggregative behavior of *B. bonariensis* has caused yield loss in infested cotton (*Gossypium hirsutum* L.) fields where heavy snail infestation covering young plants results in plant collapse. In citrus, *B. bonariensis* have caused irrigation jets to clog, resulting in foliage damage inside individual protective covers (8–10, 17, 18).

As described for other mollusks, the success of the snails in colonizing new areas may be related to high adaptability to a disturbed agricultural environment (1, 19), stimulatory effects by increased fertilizer application (20), and lack of natural enemies (1, 3). Intrinsic characteristics of the species might pose relevant contributions to its success, such as advanced defense mechanisms against stress (i.e., epiphragm formation) (21), high reproductive capacity (11, 16, 22), and low diet specificity (15).

The limited understanding of *B. bonariensis* ecology and the lack of validated management tools are critical limitations in regions with high densities of this species in row crops. Few active ingredients are available for snail management, and some formulations are not labelled against *B. bonariensis* or to be used in row crops (3, 21). Existing management strategies for snails mostly rely on chemical molluscicide pellets, containing either metaldehyde or iron (ferric) phosphate (21, 23). However, besides their high cost when used in large areas with row crops, there have been concerns over metaldehyde due to its impact on nontarget organisms and water systems (24–26). There is also a need to identify the triggers for *B. bonariensis* population growth in agricultural areas (1). For that, adoption of standard sampling and trapping methods significantly enhance the estimation of population levels and advance the development of effective snail management programs. Soil and plant inspection, bait traps, and artificial shelters are alternatives used to provide insight into relative slug populations or to control snails and slugs in small home gardens (3, 25, 27–29). However, these techniques have not yet been validated for *B. bonariensis* in commercial row crops.

Here we provide the first description of sampling, trapping, and alternative management methods of *B. bonariensis* needed for the development of an Integrated Pest Management (IPM) program of this invasive species in row crops. We also documented *B. bonariensis* dispersion capacity among row crops, and preference of crop and weed species providing shelter (referred to host-related plants). The triggers for *B. bonariensis* population growth across the southeastern U.S. are discussed.

## 2 Materials and methods

Thirty snail specimens obtained from commercial row crop fields in Santa Rosa and Escambia Counties in the Florida Panhandle were submitted for species identification to the Florida Department of Agriculture & Consumers Service (FDACS). The taxonomic analysis identified the species *B. bonariensis* (Stylommatophora: Bulimulidae).

### 2.1 Sampling

Sampling methods for *B. bonariensis* were tested during the 2019 crop season in one commercial peanut field located in Santa Rosa County, FL (30°55’58.1”N, 87°10’16.4”W). Peanut (cultivar Georgia-06G) was planted and managed based on best practices recommended by the University of Florida (30). The area had a history of snail infestation. Plant inspection, soil inspection, and beat cloth methods were tested in 38 cm wide x 1-meter long sections of peanut rows. The plant inspection consisted of a top-to-bottom examination of all peanut plants within a 1-meter row. Soil inspection was performed by examination of the soil surface near the base of the peanut plant canopy. Beat cloth sampling was conducted by placing a 1-meter-long white cloth on the ground between two peanut rows and shaking the peanut plants over the cloth. The total number of live snails was recorded in 10 replications (sampling sites) randomly selected in the 20-acre peanut field.

### 2.2 Trapping

The snail trapping study was performed during the 2019 crop season at an experimental field located at West Florida Research and Education Canter (WFREC), University of Florida, Jay, FL (30° 46’ 39.288’’ N, 87° 8’ 27.024’’ W). Peanut (cultivar Georgia-06G) was planted, and management was based on best management agronomic practices recommended by the University of Florida (30). The area had a history of snail infestation and the trapping methods tested were cardboard (‘F’ flute, brown paper, 0.8 mm thick), synthetic roofing shingles (polymer-based, black, 0.3 mm thick), beer trap, and commercial trap (snailer type, green) (Rincon-Vitova Insectaries, Inc. Ventura, CA). Both cardboard and roofing shingles were cut in a 1-m^2^ and placed on the soil surface. The area covered by the cardboard and roofing shingles was watered until moist. The beer trap consisted of a 500 ml water bottle cut in half with the top part reattached to the bottom in an inverted position. The top part had the lid removed and 200 ml of beer (Budlight, St. Louis, MO) was added to the bottle for snail attraction. The beer trap was placed in holes previously dug into the soil with a shovel. The commercial trap was used following company recommendations. The study was replicated 10 times and the number of snails per trap was recorded after 24, 48, and 72 hours.

### 2.3 Pesticide efficacy

#### 2.3.1 Cage trial

The pesticide efficacy cage trial was performed during the 2019 crop season at an experimental field located at WFREC, University of Florida, Jay, FL **(**30° 46’ 39.288’’ N**,** 87° 8’ 27.024’’ W**)**. Cylindrical metal cages of approximately 3-gallon volume were constructed with chicken wire mesh and randomly placed on the edges of a peanut field with a natural snail infestation. Metaldehyde 4% (Deadline M-Ps 4% Mini Pellets, AMVAC, Los Angeles, CA), sulfur 1% (Bug-Geta, ORTHO, San Ramon, CA), carbaryl 5% (Sevin, TechPac LLC, Palatine, IL), iron phosphate 1% (Sluggo, Neudorff, Emmerthal, Germany), and a control were tested in five replications. The control cage did not receive any chemical application. Snails were collected in infested fields, and confined in each cage before treatment. The top part of each cage was closed with pollination bags (Vilutis & Co., Inc. Frankfort, IL). The treatments (chemicals) were applied following the label recommended rates per area on each respective cage. This cage trial was arranged in a Complete Randomized Design (CRD) with 40 snails per treatment divided in four replications. Snail mortality was evaluated 72h after chemical application.

#### 2.3.2 Field trial

The pesticide efficacy field trial was conducted at WFREC, University of Florida, Jay, FL (30° 46’ 39.288’’ N, 87° 8’ 27.024’’ W), during the 2021 and 2022 crop seasons. Peanut (cultivar Georgia06G) was planted, and management was based on the University of Florida best agronomic techniques for a full-tillage system (30). There was a history of snail infestation in the area under study and the treatments included molluscicide ferric sodium EDTA 6%, 21 kg/ha (Slug & Snail Killer, OMEX Agriculture Inc., Oak Bluff, MB), iron phosphate 5%, 16 kg/ha (Ferroxx, Neudorff,Emmerthal, Germany). Based on preliminary data from insecticide bioassays performed in the Entomology laboratory at WFREC and field observations of the effect of tillage on the survival of snails, the following additional treatments were included: methomyl 3.5 ml/ha (Lannate, Corteva agriscience, Wilmington, DE), tillage, and tillage plus methomyl. The plots (4 m x 7 m) were treated with either a handheld broadcast spreader or a backpack sprayer. The tillage treatment was carried out in accordance with the recommendation for peanut cultivation in Florida (30), and control plots with no treatment application were utilized to assess the natural snail mortality during the trial. Four replications in a Randomized Complete Block Design (RCBD) were used to test treatments. Two sampling sites (1m long sections) were chosen at random from the middle four rows of each plot for plant and soil examination, and the number of live snails was recorded before and three, seven, fourteen-, and twenty-one days following plot treatment. In 2022, the same trial was repeated at the same location; however, treatments with poor snail control performance in the first year were removed. The 2022 field trial consisted of a control (fallow), tillage, tillage plus methomyl, reduced tillage, and reduced tillage plus methomyl. The treatment application and evaluation were conducted according to the previous year’s criteria.

### 2.4 Snail ecology

#### 2.4.1 Field dispersion and host-related plants

Snail field dispersion capacity was evaluated in an experiment composed of four combinations of crops cultivated side by side at the experimental area located at WFREC, University of Florida, Jay, FL **(**30° 46’ 39.288’’ N**,** 87° 8’ 27.024’’ W**)**. There were six rows in each block, with the primary crops (corn, cotton, soybean, and peanut) planted in the middle two rows. Four rows of peanuts were also planted as a border in each plot. Each crop setting was established in a plot of approximately 11 m by 11 m. Plots were separated by approximately 3.66 m alleys. The plots were planted on April 29, 2021, and the study was performed during the month of June when the crops were in the vegetative stage. Before the performance of the snail dispersion study, the entire experimental area was inspected for the presence of natural populations of snails, and no infestation was detected. *Bulimulus bonariensis* collected in commercial fields were brought to the Entomology Laboratory at WFREC and sorted considering a uniform size of adults. The method of mark-release-recapture was used and approximately 800 snails had their shell marked with neon nail polish (L.A. Girl Cosmetics, Ontario, CA) and released in the central rows of each plot. The field dispersion of the snails was evaluated two, six, 12, and 22 days after plant infestation. Evaluations were performed by ground and plant inspections in each plot (soybean, corn, cotton, and peanut). The crop origin of the snails recovered during the sampling was determined by the color code for each crop and replication adopted during the marking process, with neon nail polish. The traveled distance was calculated by Euclidean distance (31), where the distance between plants or rows is the length of the line segment connecting them (ordinary distance).

#### 2.4.2 Host-related winter weeds

The presence and abundance of snails in winter weeds were reported in the spring of 2020 (during the month of May, prior to planting season) in a 2-acre fallow field that had previously been cultivated with peanut at the WFREC, University of Florida, Jay, Florida **(**30° 46’ 39.288’’ N**,** 87° 8’ 27.024’’ W**)**. For the sampling, eight of the most abundant and predominant weed species in the region of the Florida Panhandle were chosen. Thirty plants per winter weed species were randomly selected and inspected for the presence of snails from top to bottom, including the area of the ground covered by the plant. On each plant, the number of living snails was recorded.

#### 2.4.3 Snail monitoring and weather correlation

The monitoring of snails was recorded between November 2021 and March 2022. This time corresponds to the fall, winter, and beginning of spring seasons. The field under study was located at the WFREC, University of Florida, Jay, Florida (30° 46’ 39.288” N, 87° 8’ 27.024” W). The field was previously used for peanut cultivation. A total of 16 cardboard traps (1x1 m) were randomly placed in the experimental area, and the number of living snails in each trap was recorded. At WFREC, a weather sensor measured temperature, precipitation, and humidity, which were correlated with the snails captured throughout time.

### 2.5 Statistics analysis

The snail sampling and trapping data were examined for normal distribution and homogeneity of variances before undergoing an analysis of variance (ANOVA). Cage and field pesticide trials, snail dispersion, and crop preference used as shelter data were analyzed using a generalized mixed model (GLM). Repeated measures were used when considered necessary. The snail field dispersion data were analyzed using GLM to identify differences in mean values. The monitoring data and weather influences on *B. bonariensis* were analyzed by GLM, with a negative binomial distribution. When applicable, the averages of the trials described above were separated using Tukey’s test for significant differences (*P* = 0.05). At a significance level of 0.05%, the preference of *B. bonariensis* for winter weed species was evaluated based on the non-overlapping of confidential limits. RStudio software was utilized for all statistical methods.

## 3 Results

### 3.1 Sampling

**Figure 1:**
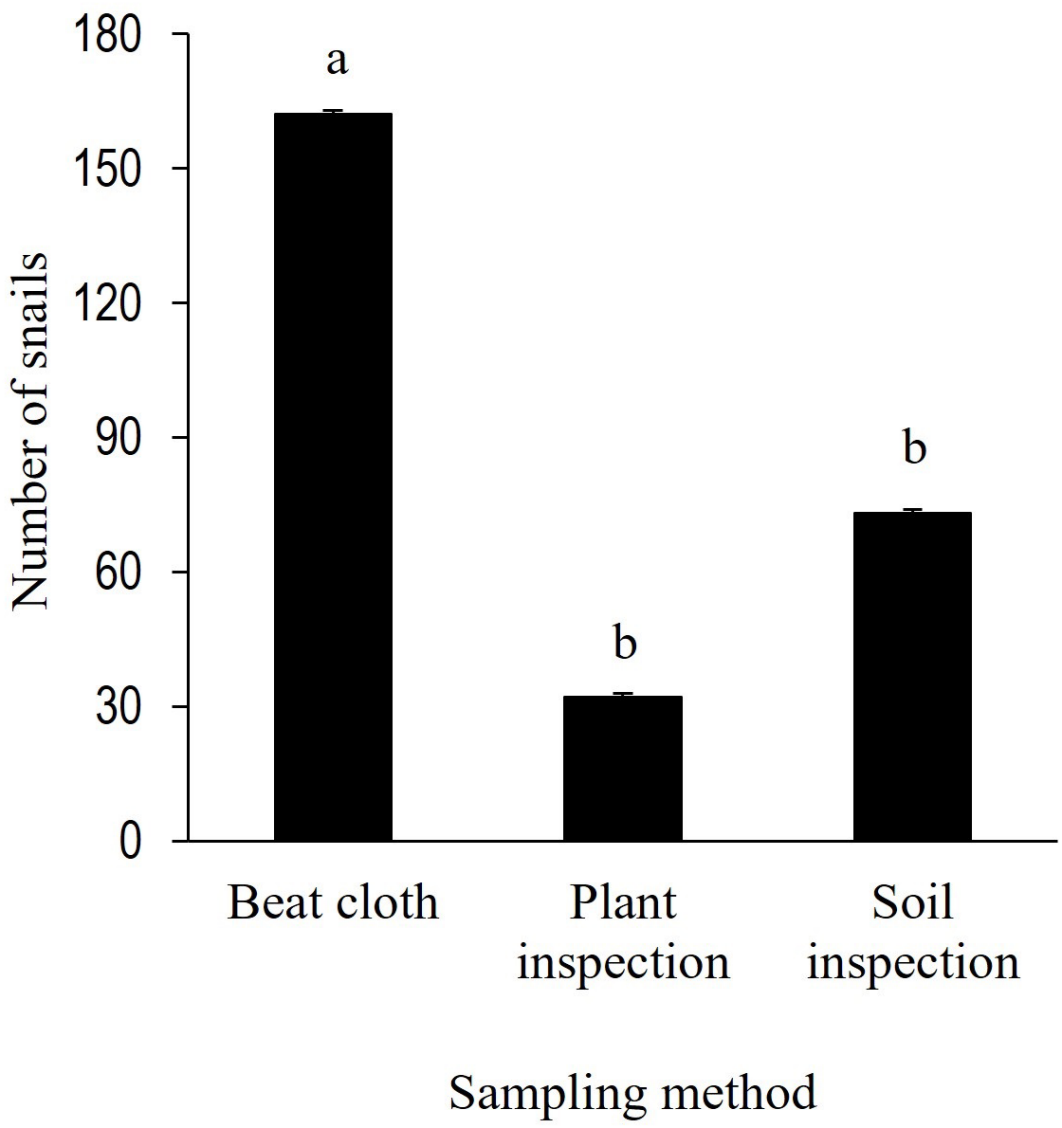
Sampling methods for snail infestation in peanut fields. Bars represent mean ± standard error. The number of *B 10.3389/finsc.2022.1056545 w/CCbonariensis* per trap was compared by one-way ANOVA with repeated measurements (*P* < 0.05).

Among the snail sampling methods tested, the use of beat cloth recorded the highest levels of snail infestation, which was ~ 122% greater than soil inspection and ~ 406% greater than plant inspection. Levels of snail infestation in soil inspection and plant inspection were not significantly different (**Figure 1**).

### 3.2 Trapping

**Figure 2:**
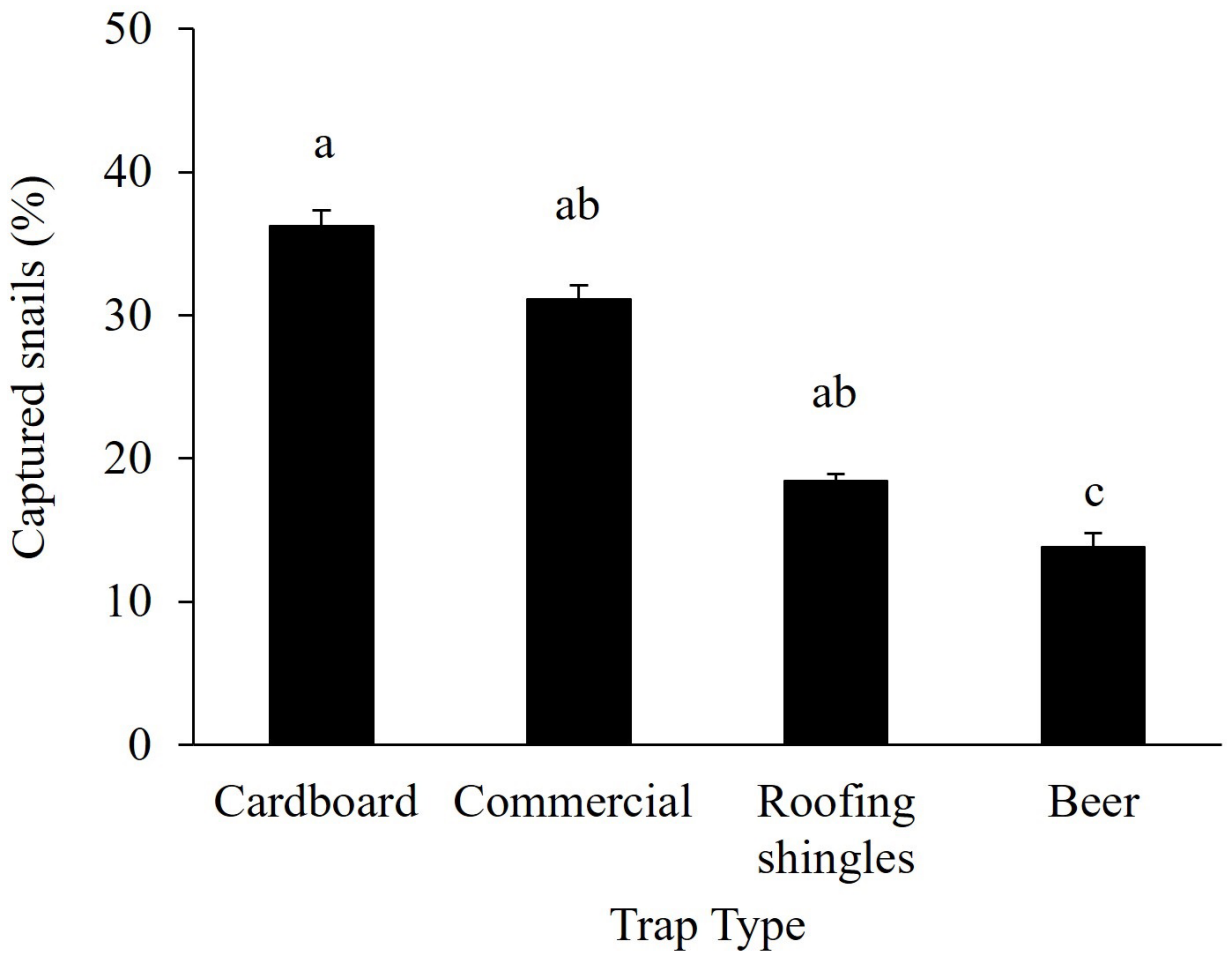
*Bulimulus bonariensis* captured in different types of traps in peanut field. Bars represent mean ± standard error. The number of snails per trap was compared by one-way ANOVA followed by the Tukey HSD *post hoc* test. Different letters indicate significant differences among traps (*P* < 0.05).

Among the *B. bonariensis* trapping methods tested, cardboard traps had a higher number of snails (36%), which was significantly greater than the snails captured in beer traps (14%), but not significantly different than for roofing traps (19%) and commercial traps (31%) (**Figure 2**).

### 3.3 Pesticide efficacy

#### 3.3.1 Cage trial

**Figure 3:**
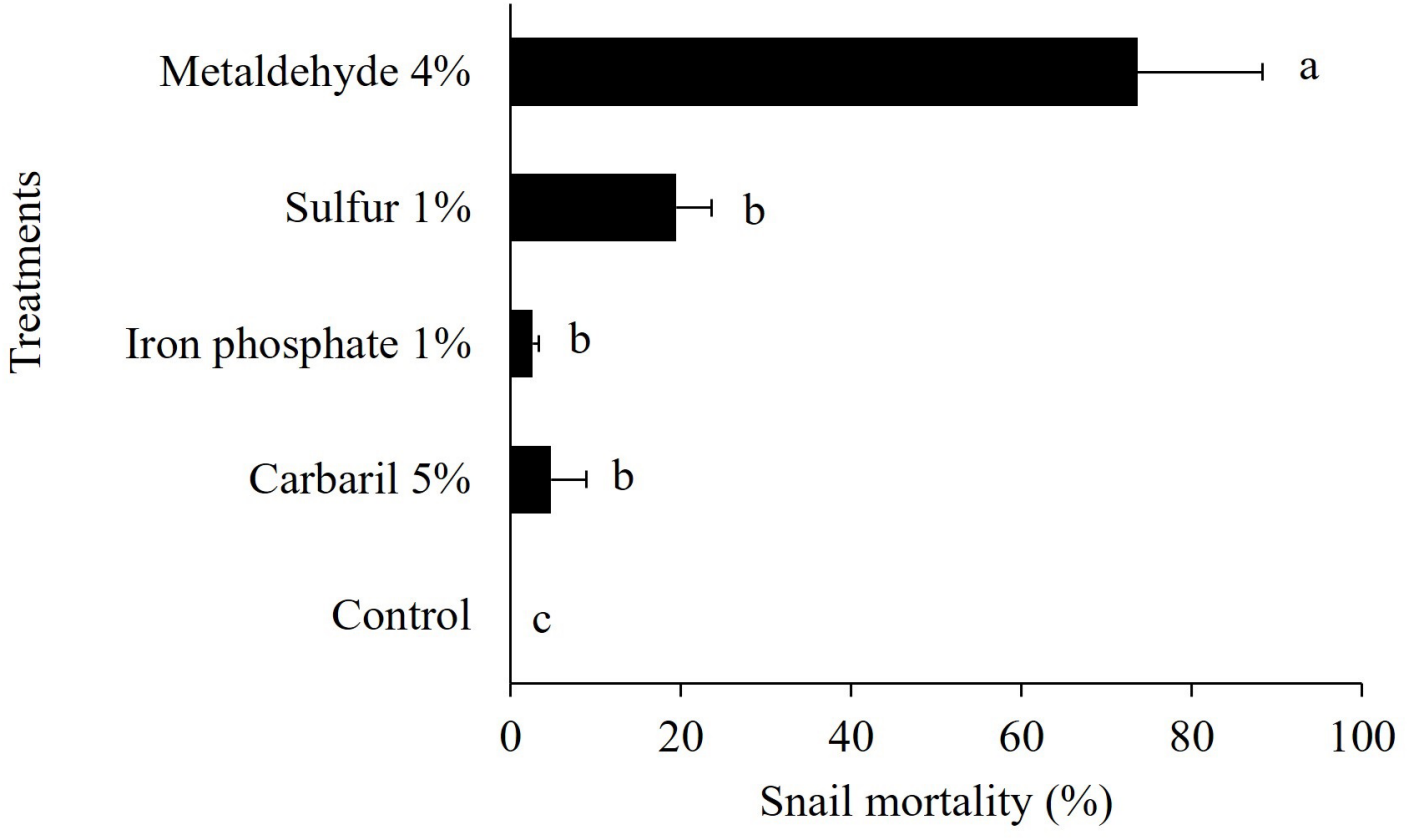
*Bulimulus bonariensis* mortality with different chemical control products. Bars represent mean ± standard error. Bars with different letters are significantly different from other treatments (P ≤ 0.05, Tukey test).

Metaldehyde 4% was the pesticide that caused the highest mortality (74%) for snails confined in metal cages. Sulfur 1% (19%), iron phosphate (3%), and carbaryl 5% (5%) did not reach 20% mortality and were significantly lower than metaldehyde (**Figure 3**). The control treatment, without pesticide had no snail mortality.

#### 3.3.2 Field trial

**Figure 4:**
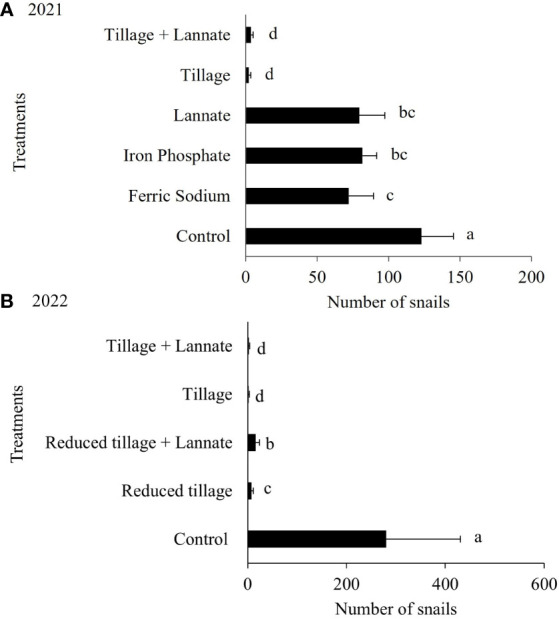
Total number of live snails sampled during the **(A)** 2021 (z= 14.96 *P*< 2e-16) and **(B)** 2022 (z= 8.122 *P*= 4.57e-16) field trials. The number of *B bonariensis* was recorded weekly for four weeks after treatment applications. Data were analyzed using repeated measures analysis of variance and a binomial negative GLM. Bars followed by the same letter are not significantly different (Tukey test at 0.05% significance).

During the field trial conducted in 2021 and 2022, tillage treatments alone or in combination with insecticide had the lowest number of living snails compared to the other treatments. During the snail field trial in 2021, tillage treatments reduced snail populations more than the control, iron phosphate, and methomyl alone (**Figure 4**). The ferric sodium-treated plots had fewer snails than the control plots, but were comparable to those treated with iron phosphate and methomyl alone. In 2022, fewer snails were found in plots with tillage or reduced tillage compared to the control (fallow area) (**Figure 4**). However, the reduced tillage plus methomyl (i.e., Lannate) treatment had more snails than the tillage treatment.

### 3.4 Snail ecology

#### 3.4.1 Field dispersion

**Figure 5:**
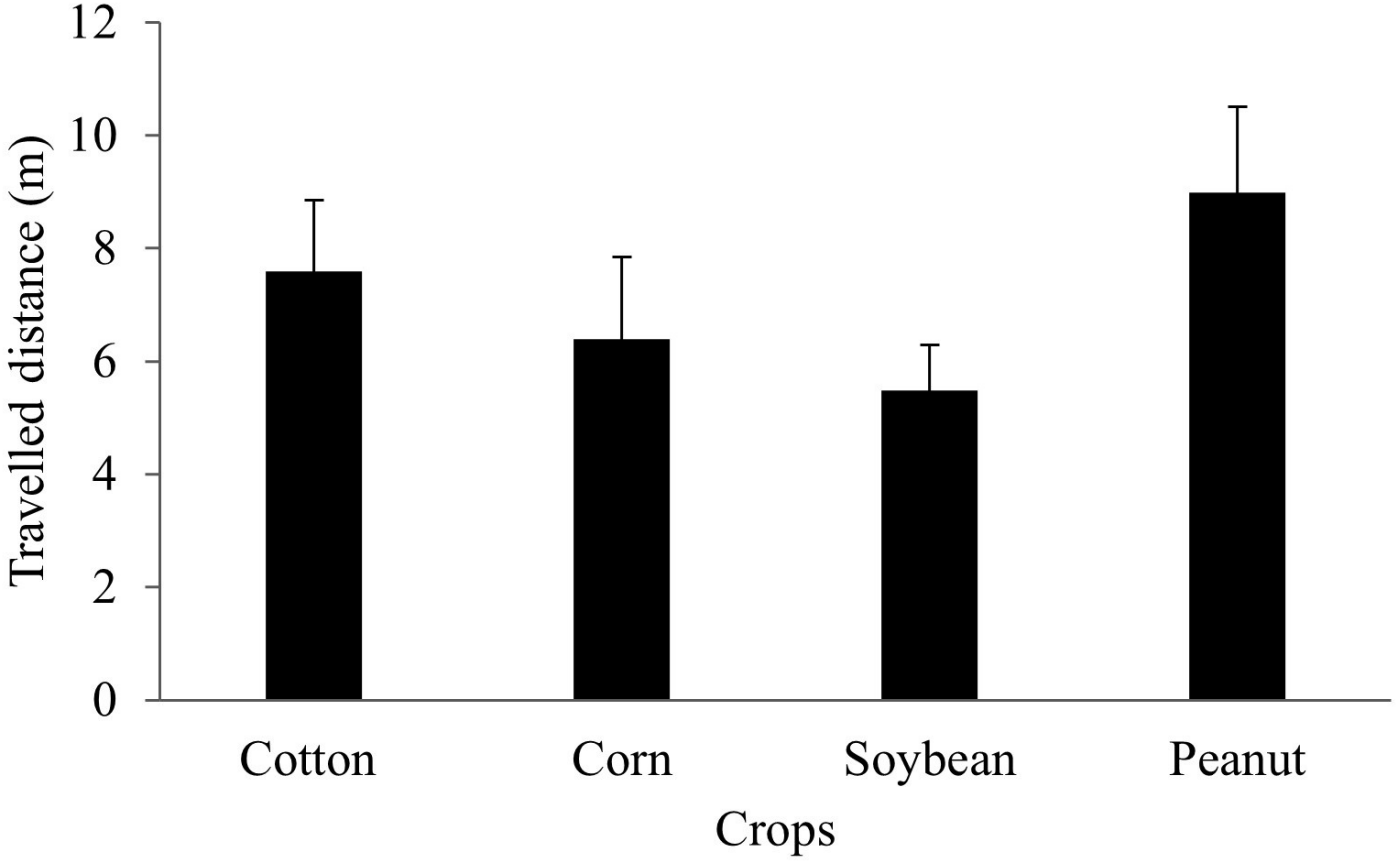
Euclidean distance (i.e., field dispersion) traveled by marked *B bonariensis* released in cotton, corn, soybean, or peanut. Bars represent mean ± standard error. Distance traveled by snails from the release crop was compared by one-way ANOVA (*P* > 0.05).

There was no statistical difference in the estimated Euclidean distance traveled by marked *B. bonariensis* released in different row crops (*P* > 0.05) (**Figure 5**). Two days after infestation, marked snails released in corn, cotton, peanut, and soybean plots traveled an average of 1 m. Twenty-two days after release of the marked snails, the further travelled distance detected was 21,8 meters.

#### 3.4.2 Host-related plants

**Figure 6:**
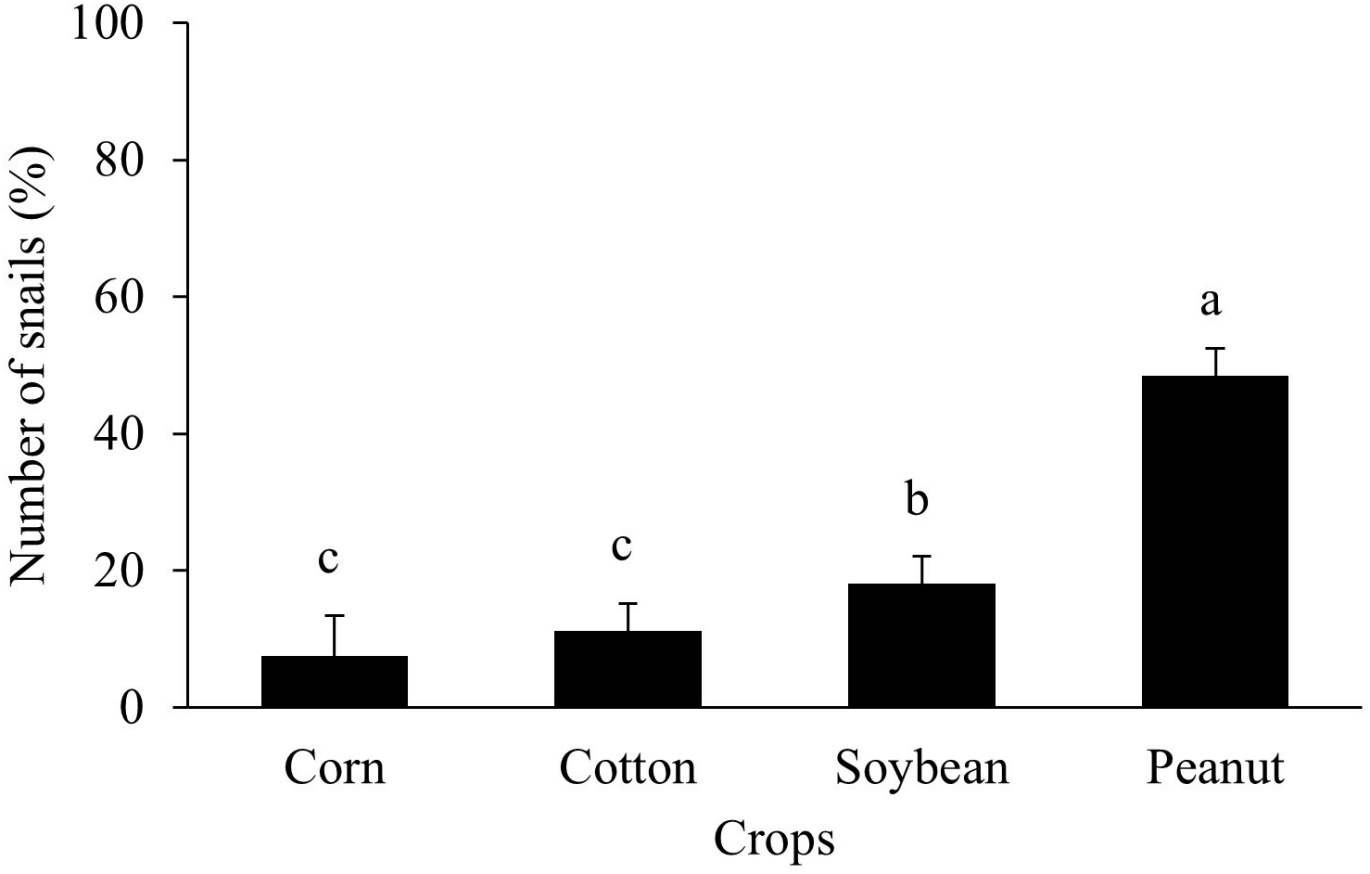
Percentage of marked *B bonariensis* recovery in each row crop. Bars are the number of snails in each crop ± standard error. Different letters indicate significant differences in snail number (*P* < 0.05, Tukey test).

Out of the 800 marked snails released in peanut, soybean, corn, and cotton, 366 snails were recovered among the four crops (**Figure 6**). A significantly high number of the snails (57%) were recovered in peanut plots, followed by soybean (21%) (*P* > 0.05). There was no difference in the number of snails recovered from cotton (13%), and corn (9%).

#### 3.4.3 Host-related winter weeds

Among the eight non-crop host plants inspected in the fallow area, cutleaf primrose and dandelion had the highest average of 7.27 and 11.27 snails per plant, respectively, followed by desertchicory (3.67), purple cudweed (3.47), southern rockbell (2.57), and clover (1.73). Wild radish (1.40) and burn weed (0.6) had the lowest number of snails among all plant species (**Table 1**).

**Table 1:** Mean number of *B. bonariensis* detected in predominant winter weeds with spontaneous growing during fallow season, in area previously cultivated with peanut.

Scientific name	Botanic Family	Common name	Mean number of snails per plant	SE^*^
*Oenothera laciniata*	Onagraceae	cutleaf primrose	11.27 a	± 3.42
*Taraxacum officinale*	Asteraceae	dandelion	7.27 a	± 1.99
*Pyrrhopappus carolinianus*	Asteraceae	desert-chicory	3.67 ab	± 0.76
*Gamochaeta purpurea*	Asteraceae	purple cudweed	3.47 ab	± 1.32
*Wahlenbergia marginata*	Campanulaceae	southern	2.57 ab	± 1.22
*Trifolium repens*	Fabaceae	rockbell clover	1.73 b	± 0.51
*Raphanus raphanistrum*	Brassicaceae	wild radish burn	1.40 b	± 0.60
*Erechtites hieraciifolius*	Asteraceae	weed	0.67 b	± 0.26

^a–b^ Means within a row with different superscripts differ (P < 0.05). ^*^ Standard Error (SE).

#### 3.4.4 Snail monitoring and weather correlation

**Figure 7:**
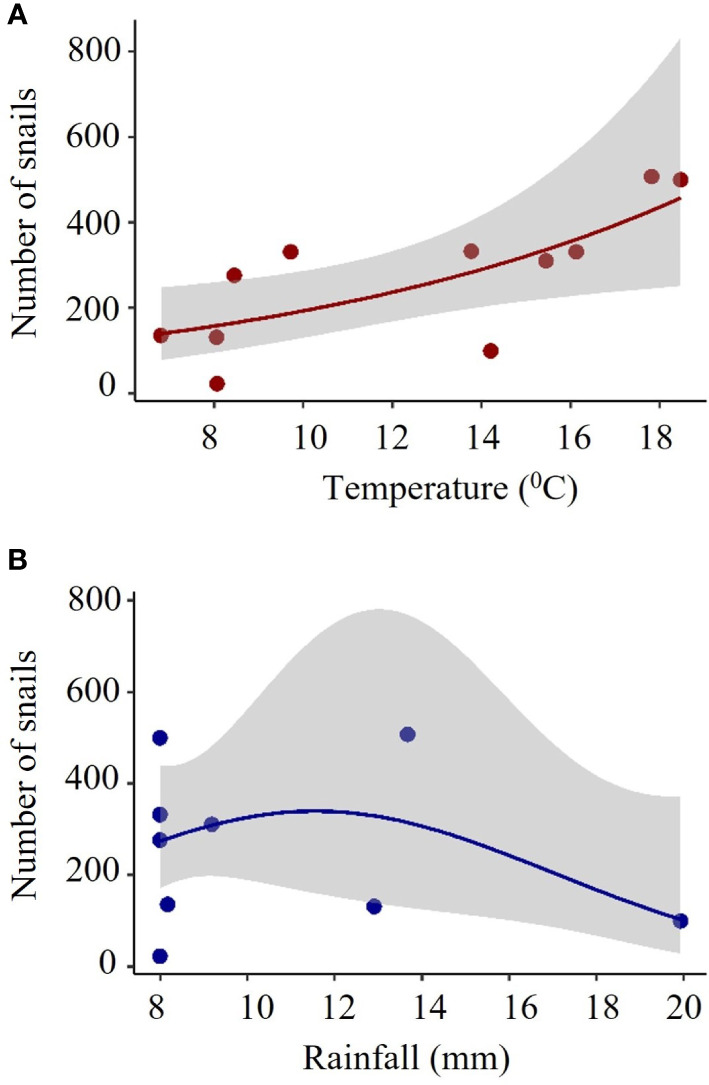
Total number of *B bonariensis* relative to **(A)** temperature and **(B)** rainfall. Snails were trapped in 16 cardboard traps over five months, including fall, winter, and spring (November 2021 to March 2022). The red and blue lines indicate the model GLM. with a negative binomial distribution. The gray area indicates the confidence of intervals. Each full red or blue circle represents the total number of snails collected in 16 cardboard traps (total number of observations is 176). Temperature: Z= 3.207 *P*= 0.00134. Rainfall: Z= -2.063 *P*= 0.03910.

The recorded number of alive *B. bonariensis* was correlated with temperature and rainfall, but not with humidity. During the trapping period of this study, the number of alive snail counted under the cardboard traps tend to increase following the increase of the temperature to 18 degrees Celsius. In contrast, intense rainfall of around 20 mm had a negative effect on the total number of snails captured (**Figure 7**).

## 4 Discussion

**Figure 8:**
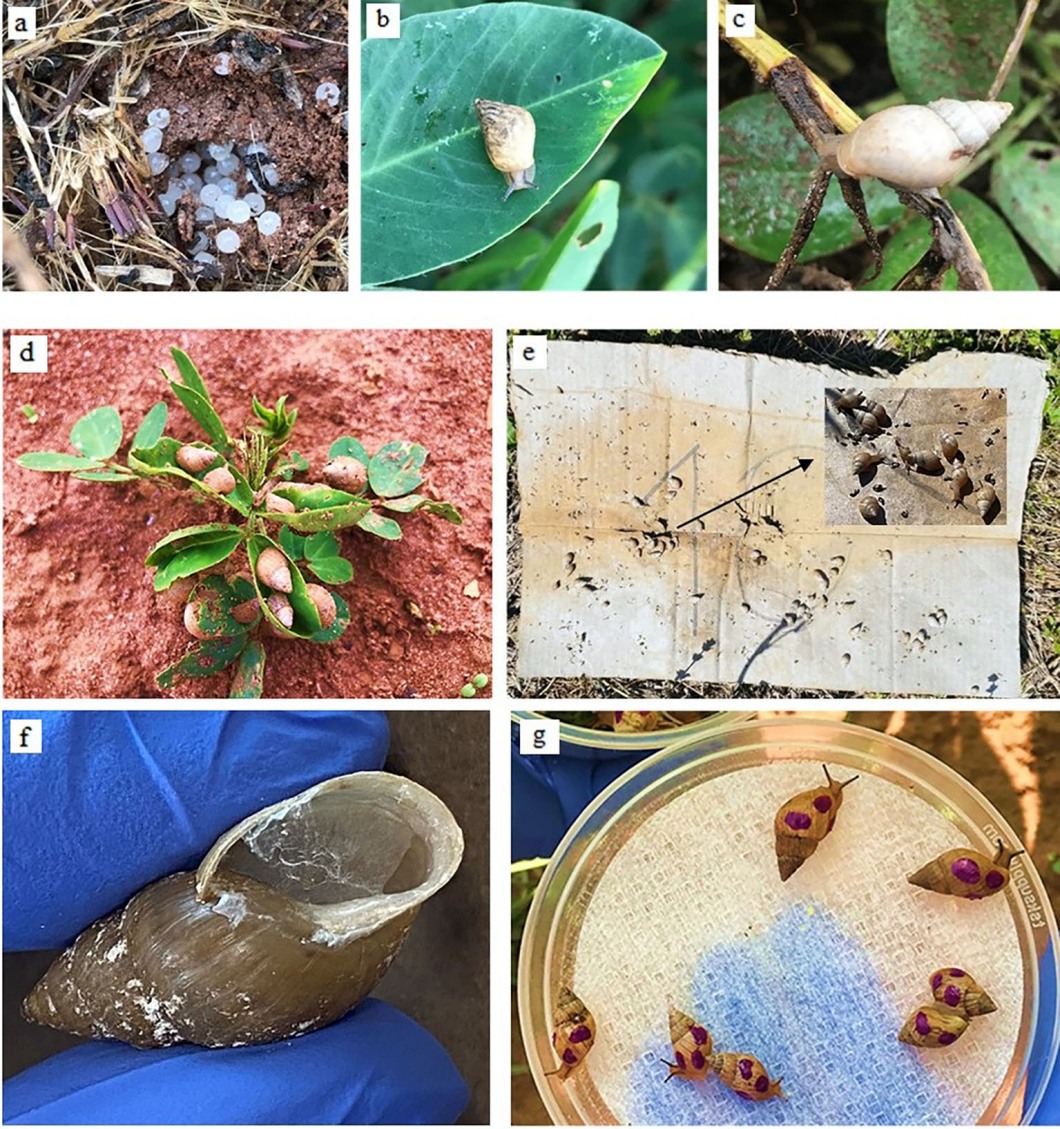
Infestation of peanut crops by *B bonariensis* eggs **(A)**, juveniles **(B)**, and adults **(C)**. Aggregative behavior of *B bonariensis* on peanut plants **(D)**. Cardboard used as snail trap **(E)**. Mucus and epiphragm in *B bonariensis* **(F)**. Snails marked with nail polish in dispersion and host preference field trials **(G)**. Adult snail length is approximately 2.5 cm. UF/IFAS, WFREC, Jay, FL.

This study provides information on the ecology of the invasive species *B. bonariensis* and validation of its control techniques, with a focus on peanut (**Figure 8**). Integrated Pest Management programs rely on the detection and quantification of pest abundance as one of its foundations. Sampling with beat cloth yielded the greatest snail counts and was a more efficient method for estimating snail infestation than searching for snails in the soil or plant inspection. Cardboard was the most effective trapping material compared to commercial traps, roofing shingles, and beer. The majority of the snail and slug traps that were tested in this research are already widely used as an alternate method of catching snails and slugs that cause damage to agricultural areas and household gardens (3). Similarly, the use of beer as bait inside of a plastic bottle is a technique that is commonly employed for slugs (29). Although the other snail traps captured a substantial number of snails, bait traps (beer and commercial)can be expensive to be adopted in large areas with row crops and be washed away or diluted by rain. In the same way, roofing shingles become excessively hot on sunny days (3, 29). Besides being a cheaper trapping method, cardboard absorbs and releases moisture, which creates favorable habitat for snails by preserving moisture and decreasing soil temperature and light. Snails found under a cardboard piece could be simply crushed and buried.

Overall, most of the pesticides in the cage study had poor performance (< 80% of control). The chemical formulation of molluscicides is limited, and despite efforts to create novel formulations for managing slugs and snails in agriculture, little progress has been made (32, 33). Metaldehyde 4% was a more effective pesticide than carbaryl 5%, iron phosphate 1%, sulfur 1%, and ferric sodium 1%. However, according to the Environmental Protection Agency (U.S. EPA), this product is not labeled for use in peanut, likely due to its toxicity. Since the 1940s, metaldehyde has been utilized; it is water-soluble, highly mobile in soils, and generally resistant to abiotic degradation. This represents a real challenge for the management of *B. bonariensis* in peanut. In addition, alternative chemicals to manage snails can be compromised by the physiological and behavioral traits of land mollusks. In general, these organisms can present estivation, retraction of the cephalopod mass, and burying, which can promote survival following exposure to molluscicides (21). In the present study, increased mucus secretion and epiphragm development, which are used to seal the shell aperture were frequently observed in the pesticide trials, which partially explains the observed variation in management efficacy among treatments.

To ensure pesticide efficacy for snail management, it is necessary to synchronize chemical management with periods of snail activity. However, this occurs during rainy periods when agriculture machinery has limited access to wet fields and the bait efficacy period is shortened. Based on these limitations, tillage was included as an alternative cultural control to snails, prior to peanut planting. Tillage alone or in conjunction with an insecticide methomyl indicated the most efficient snail management strategy (>80% mortality). The tillage can potentially kill large number of snails that would be a source for initial infestations. Tillage also removes crop residues and ground cover, as their presence certainly provide a favorable environment for snails. However, this strategy alone might not be enough to contain snail populations in long term, especially if the surround areas remain infested. Tillage might contradict the soil conservation principles (34) and should be considered in areas with historical snail infestation and where erosion is less of a concern (3).

A significant obstacle is that little is known about the ecology of *B. bonariensis*, especially its lifetime and oviposition parameters. Observations during this study indicated that eggs are laid on the soil beneath the peanut canopy. Soon after hatching, snail must construct a protective shell; in some species, parental efforts include providing calcium-rich feces or coating the eggs with a layer of calcium-rich dirt (20, 35). This behavior has been reported in the invasive garden snail, *Helix aspersa* (Müller, 1774), which egg production, was almost doubled in the second part of the trial when calcium carbonate was added to calcium-deficient soil (36). In this way, calcium-based fertilization may be a relevant aspect and should be in the future investigated and be considered in an IPM program for snails in row crops. In the southeastern United States, heavy calcium-based fertilization (up to 2241 kg/ha) is commonly adopted by peanut growers. Calcium not only promotes aboveground vegetative plant growth, but also contributes directly to fruit development below the soil surface, hence improving crop production (20, 37, 38). High calcium fertilization in peanut and other crops may be one of the reasons why initial small population of the invasive *B. bonariensis* have increased in recent years, particularly in peanut-producing regions. *Bulimulus bonariensis* may be better adapted to the biotic and abiotic conditions in the current intensified agricultural landscape, aligned with very low competition from other snail species and a low number of natural enemies such as carabid beetles and nematodes, which is a classic scenario during biological invasions (1, 19). Future studies should focus on the impact of calcium-based fertilization in the ecology of *B. bonariensis.*

In Florida peanut-producing regions, the presence of *B. bonariensis* was documented in eight winter weed species that infest areas during the fallow season. Cutleaf primrose supported an average of 11.27 snails per plant, which is 55% more than dandelion, the second-highest host-related winter weeds species with snail. We have reported an overall dispersion distance of 10m for individuals released in maize, cotton, peanut, and soybean crops, with no observed differences in dispersion distance between crops. The weather appears to be the primary external element driving the movement, with the majority of movement occurring on mild days shortly after moderate rain. In the present study, the effect of the humidity was not detected, which should not be irrelevant in snail ecology, but because it was constant and high in all months during the performance of the study.

Peanut was a host-related crop for snails (54%), chosen over other row crops as a preferred shelter. Little is known about each internal or external factor influencing the distribution of *B. bonariensis*, but typically, organisms foraging in regions with rich resources move slowly (39). Peanut prostate canopy growth most likely provides superior shelter since snail mobility is driven by local environmental circumstances among other factors (39).

This study validated IPM tools for the invasive snail *B. bonariensis*. We determined that beat cloth and cardboard traps are effective tactics to be used to detect and monitor *B. bonariensis* infestations in peanut and other row crops. Currently, peanut farmers in the southern United States have few molluscicide options. The use of methomyl may be considered and restricted to be adopted during soil preparation in situations of high snail infestation, due to its risk to natural enemies during the peanut crop season. In addition, tillage is indicated to be a viable method for managing juvenile and adult snail infestations, while additional research is required, especially in view of the growing reliance on conservation tillage methods. The weather appears to be the driving force behind snail field dispersal in row crops, and the prostrate growth habit of peanut provides a favorable environment for *B. bonariensis*, making this crop a preferred habitat. Additional research on the biology and behavior of *B. bonariensis* where calcium-based fertilized are used is necessary to increase the efficacy of management programs and contribute to a greater understanding of this invasive pest since its occurrence range has expanded and become a concern to the economy and food security.

## Data availability statement

The raw data supporting the conclusions of this article will be made available by the authors, without undue reservation and upon request.

## Author contributions

All authors have contributed equally within specific sections of this manuscript. SP-M designed and secured funding for the research. MM and MD conducted the experiments, analyze the data, and wrote the first draft of the manuscript. SP-M performed reviews, edits, and consolidated the final version of the manuscript.
